# Partial Anomalous Left Pulmonary Artery Anterior Versus Posterior Types: A Systematic Review

**DOI:** 10.3390/tomography8040163

**Published:** 2022-07-27

**Authors:** Carlos S. Restrepo, Tomas V. Gonzalez, Ameya J. Baxi, Sachin S. Saboo

**Affiliations:** 1 Department of Radiology, UT Health San Antonio, 7703 Floyd Curl Dr, San Antonio, TX 78229, USA; restrepoc@uthscsa.edu (C.S.R.); baxi@uthscsa.edu (A.J.B.); saboo100@gmail.com (S.S.S.); 2 Department of Radiology, Mayo Clinic, 200 1 St SW, Rochester, MN 55905, USA

**Keywords:** partial anomalous left pulmonary artery (PALPA), pseudopulmonary artery sling, duplicated left pulmonary artery, congenital heart disease, Kabuki syndrome

## Abstract

The aim of this study was to investigate the features of partial anomalous left pulmonary artery (PALPA) and differences between cases with posterior versus anterior a nomalous vessels in relation to the tracheobronchial tree. We hypothesized that statistical significance was dependent on the course of the anomalous vessel due to airway compression in the posterior type. This study included cases obtained from the literature (*n* = 33) and an institution teaching file (*n* = 2). Information collected: age, sex, medical history, additional anomalies, anomalous vessel course, and respiratory symptoms. Data were analyzed with independent samples *t*-test and Fisher’s exact test. PALPAs were more commonly anterior than posterior. Mean age: 5.3 years (SD = 12.4) for anterior and 6.8 years (SD = 18.5) for posterior (*p* = 0.77). Respiratory symptoms: 20% of anterior and 60% of posterior cases (*p* = 0.032). Tracheobronchial anomalies: 35% of anterior and 60% of posterior cases (*p* = 0.182). Non-cardiac and non-tracheobronchial anomalies: 30% of anterior and 47% of posterior cases (*p* = 0.511). Kabuki syndrome: 25% of anterior and 6.7% of posterior cases (*p* = 0.207). In conclusion, respiratory symptoms were the only significant difference between anterior and posterior PALPA types.

## 1. Introduction

Partial anomalous left pulmonary artery (PALPA) or duplicated left pulmonary artery (LPA) is a very rare congenital vascular anomaly. It is characterized by an arterial branch arising from the right pulmonary artery (RPA) and feeding the left lung, in addition to the presence of proper LPA arising from the main pulmonary artery in a normal fashion resulting in a duplicated LPA [1]. Most PALPAs terminate in the left lower lobe. Three types of PALPA have been described based on their relationship with the tracheobronchial tree. These include the anomalous vessel arising from the inferior proximal right PA and passing anterior, antero-inferior or posterior to the tracheobronchial tree with the latter type forming a Partial PA sling or PALPA “sling” [2,3]. This anomaly must not be confused with a left pulmonary artery sling (LPAS)/Typical pulmonary artery (PA) sling or aberrant origin of the LPA [4]. There is a known association between PALPA and Kabuki syndrome (also known as Niikawa–Kuroki syndrome), a rare congenital anomaly in which multiple malformations coexist: characteristic facies, skeletal anomalies, dermatoglyphic abnormalities, intellectual disability and growth retardation [3,5].

Similar to what is the case with pulmonary artery sling, different tracheobronchial malformations including right upper lobe bronchus, bridging bronchus and tracheobronchial stenosis, as well as congenital heart disease, skeletal, gastrointestinal, and genitourinary malformations have been reported in PALPA patients. Current literature on this rare pathology is limited to single case reports and small case series. The purpose of this study was to systematically collect the largest collection of PALPA cases from the literature and a single teaching institution to help better delineate the unique clinical and radiologic features of this rare entity and further assess differences between subtypes according to their anterior versus posterior relationship to the tracheobronchial tree. 

## 2. Materials and Methods

**Figure 1:**
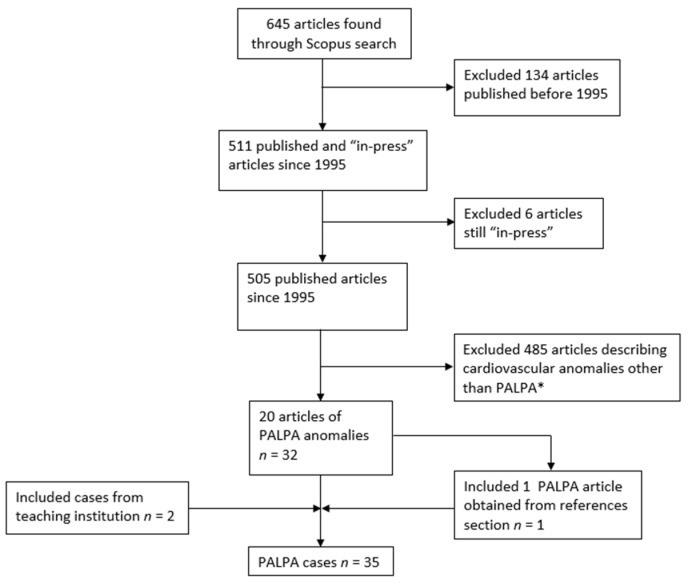
Flowchart depicting systematic article exclusion and overall PALPA case acquisition from the literature and a single teaching institution. * = including but not limited to partial anomalous pulmonary venous connection, left pulmonary artery sling and anomalous coronary artery connection.

The literature was searched for cases of PALPA using the Scopus database and following the PRISMA criteria (Figure 1). This study was Health Insurance Portability and Accountability Act (HIPAA) compliant and Institutional Review Board (IRB) exempt. The following terms were used on the search query: “partial anomalous left pulmonary artery” or “duplicated left pulmonary artery” or “pseudopulmonary artery sling” or “pseudopulmonary” or “partial pulmonary artery sling” or “left pulmonary artery sling” or “subcarinal pulmonary artery sling”. No restrictions on the basis of publication language were made. Articles in press and those published before 1995 were excluded as virtually all cases with definite imaging confirmation were published after this year. Patients with aberrant LPA either posterior to the trachea (Pulmonary artery sling), or anterior to the trachea, but without duplication of the LPA were also excluded. The reference sections of all relevant articles were scrutinized for additional cases. The resulting cases were combined and analyzed in conjunction with those from the teaching files of a single teaching institution. The following data were collected when available: age, sex, respiratory symptoms, past medical history, presence of Kabuki syndrome, associated tracheobronchial anomalies, anomalous arterial course, concomitant congenital heart disease and any additional non-cardiopulmonary malformation. All cases were reviewed by two board-certified radiologists with more than twenty years of combined experience in cardiothoracic radiology. 

Data were analyzed using the IBM SPSS statistics (Version 27) predictive analytics software. In deciding between the Chi-squared test and the Fisher’s exact test for the analysis of categorical variables, our relatively small sample sizes (less than 30 for each cohort of anterior versus posterior PALPA) made the Fisher’s exact test the most accurate and appropriate test for the present study. To facilitate data analysis, PALPA with an anterior and anterior–inferior course were grouped together. Categorical variables analyzed included presence or absence of respiratory symptoms, tracheobronchial anomalies, left lower lung supply, cardiovascular anomalies other than PALPA, anomalies not of cardiovascular or tracheobronchial origin, and a reported diagnosis or suspicion of Kabuki syndrome. To allow for analysis, all categorical variables were codified with binary numerical values representing presence of absence of said variable (e.g., 1 = presence, 0 = absence) in the statistical software. Age represented the single continuous numerical variable analyzed via a two-sample t-test. Given that different publications reported age in different units (e.g., days, weeks, months, years), all ages were converted to days using the conversions 1 year = 365 days, 1 month = 4 weeks, and 1 week = 7 days to allow for the appropriate pooling of data.

## 3. Results

The Scopus database search yielded 645 articles that resulted in 21 articles describing 33 individual cases of PALPA after application of the inclusion and exclusion criteria. These 33, in addition to two cases from a single participating teaching institution, resulted in a total of 35 cases (Figure 1). Anterior PALPA were a little over half of all cases (20/35; 57.1%) with the rest being posterior (15/35; 42.9%). Please refer to Table 1 for detailed findings per individual case. Anterior PALPA had a mean age of 5.3 years (SD = 12.4) while posterior type had a mean age of 6.8 years (SD = 18.5) (*p* = 0.77). Sex was reported in 16/20 of anterior PALPA patients, with 10/16 (62.5%) being male; similarly, 10/15 of posterior PALPA patients had the reported sex with 6/10 (60%) being male. Respiratory symptoms were reported in 4/20 (20%) of anterior PALPA and included “issues with airway”, “cough” × 2, and “shortness of breath” while 9/15 (60%) of those with posterior PALPA had respiratory symptoms which included “increased work of breathing”, “dyspnea/dyspnea on exertion” × 2, “stridor” × 2, “pulmonary infections and cough”, “pulmonary infections and pulmonary hypertension”, “cough and shortness of breath” and “severe respiratory distress” (*p* = 0.032). Tracheobronchial anomalies were reported in 7/20 (35%) of anterior and 9/15 (60%) of posterior PALPA patients (*p* = 0.182). The anomalous vessel was reported to feed exclusively the left lower lung in 15/20 (75%) cases of anterior and 10/15 (66.7%) of posterior PALPA (*p* = 0.712). Cardiovascular anomalies other than PALPA were reported in 18/20 (90%) of anterior and 9/15 (60%) of posterior cases (*p* = 0.051). Anomalies not of cardiovascular or tracheobronchial origin were reported in 6/20 (30%) of anterior and 7/15 (46.7%) of posterior cases (*p* = 0.481). Confirmed or suspected Kabuki syndrome was reported in five cases of anterior (25%) and in only one case of posterior (6.7%) PALPA (*p* = 0.207).

## 4. Discussion

**Figure 2:**
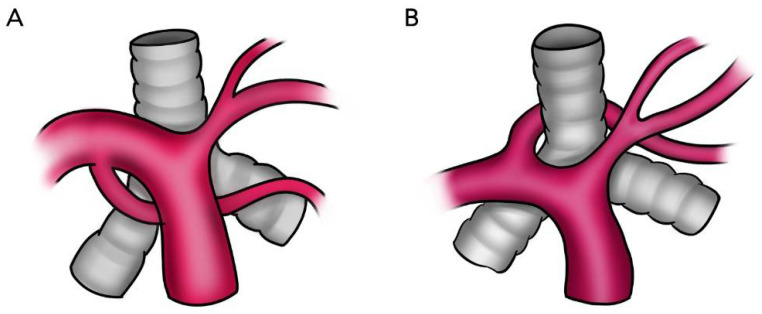
Schematic diagram of two representative patterns of PALPA branching and their anatomic relation with the tracheobronchial tree. (**A**) The anomalous duplicated left pulmonary artery originates from the inferior aspect of the right pulmonary artery and passes to the left anteriorly to the tracheobronchial tree. (**B**) The anomalous duplicated left pulmonary artery arises from the posterior–superior right pulmonary artery and passes to the left hilum between the trachea and the esophagus (not shown).

**Figure 3:**
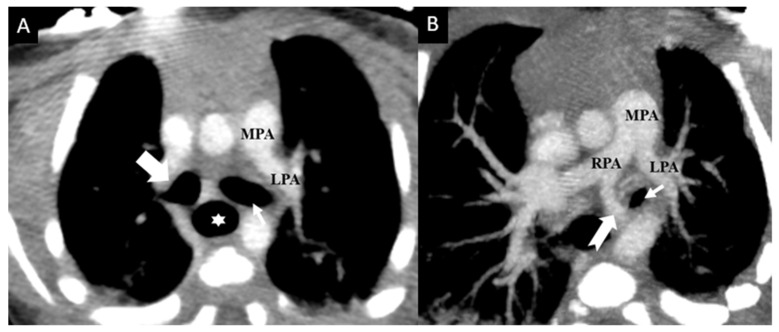
Newborn male with complex congenital heart disease (double outlet right ventricle, ventricular septal defect, and aortic coarctation) with PALPA. Contrast enhanced axial (**A**) and (**B**) axial oblique reconstructed Maximum Intensity Projection (MIP) images from chest CT data demonstrate a duplicated left pulmonary artery with a normally arising left upper lobe pulmonary artery (LPA) from the main pulmonary artery (MPA) coursing anterior to the left main bronchus (thin small arrow), while an anomalous left lower lobe pulmonary artery (notched arrow) coursing inferior anterior to the left main bronchus and left lateral to the esophagus (star). No anomalous vessel was seen between the trachea and the esophagus. Right main bronchus is marked with a bold white arrow.

**Figure 4:**
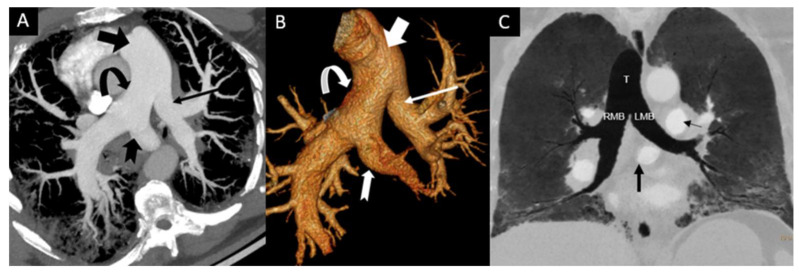
Incidental finding of PALPA in a 51-year-old male with acute shortness of breath. Contrast enhanced chest CT obtained to rule out pulmonary embolism demonstrates that a duplicated left pulmonary artery with a small left pulmonary artery (thin arrow) to left upper lobe was arising from the main pulmonary artery (bold arrow) and an additional anomalous left pulmonary artery to the left lower lobe (notched arrow) was arising from the proximal posterior part of the right pulmonary artery (curved arrow) on axial oblique reconstructed Maximum Intensity Projection (MIP) image (**A**) and Volume Rendered reconstructed image (**B**). There was no associated congenital heart disease or tracheobronchial stenosis. Coronal Minimum intensity projection (MinIP) (**C**) reconstructed image from same patient shows that the course of anomalous LPA branch (PALPA, bold black arrow) is caudal to the carina and is inferior anterior and parallel to the left main bronchus (LMB). There was no tracheal (T), right main (RMB) or left main bronchial (LMB), or esophageal compression. Note that left upper lobe and the lingula were supplied by the proximal left pulmonary artery (thin black arrow) which arises in the usual position from the main pulmonary artery (MPA). Note peripheral reticular opacities and ground glass opacities in both lungs related to known non-specific interstitial pneumonia (NSIP).

**Figure 5:**
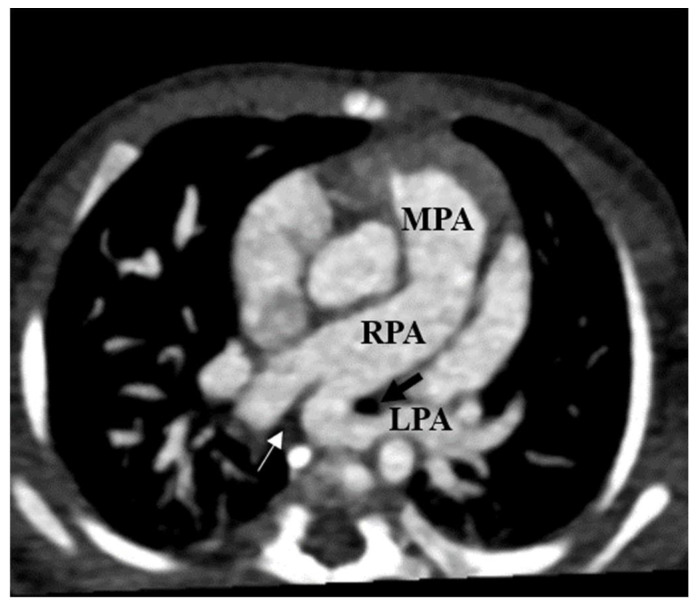
Left pulmonary artery sling (LPAS) in a 3-month-old girl with a cardiac murmur. Axial thin chest CTA shows an anomalous left pulmonary artery (LPA) arising from the posterior part of the proximal right pulmonary artery (RPA) and coursing between the left main bronchus (black arrow) and esophagus (thin white arrow) to the left pulmonary hilum.

LPA abnormalities can be distinguished by their relation to the tracheobronchial tree, the presence of native LPA, an anomalous origin and suspected airway compression. In patients with PALPA, the accessory LPA may have a variable course, including between the tracheobronchial tree and the esophagus or may run either anterior or antero-inferior to the tracheobronchial tree (Figure 2). This anomalous branch of the RPA provides vascular supply to the left lung, in addition to the normal LPA, which originates from the typical bifurcation of the main pulmonary artery (Figure 3, Figure 4 and Figure 5). Regardless of its course, our findings support the previously reported predilection to supply the left lower lobe [3]. PALPA associated with a posterior course was found in 15/35 cases (42.9%) which is increased from prior reports (22.2%) [3]. Another possible pulmonary artery anomaly is the aberrant origin of the LPA from the RPA in which the anomalous LPA is anterior or inferior to the trachea but without duplication, hence not a LPAS and not PALPA and is thus called an aberrant LPA without a sling. Similarly, in such cases, an association with congenital heart disease, and congenital tracheobronchial anomalies have been described [24,25,26].

PALPA and other pulmonary artery abnormalities are thought to be related to developmental defects of the sixth aortic arch [1,18,27,28]. On the 29th day of embryonic development, the aortic sac gives rise to the ventral bud of the sixth aortic arch and fuses with the simultaneously developing dorsal bud of the sixth aortic arch arising from the dorsal aorta; this connection gives rise to the complete sixth aortic arch proper [18,27,28]. The ventral bud of the sixth aortic arch sends a branch to the primitive lung vasculature, otherwise known as the post-branchial plexus forming the pulmonary artery [18,27,28]. The dorsal bud of the right sixth aortic arch disappears while the left persists as the ductus arteriosus [18,27,28]. If the ventral bud of the left sixth aortic arch and left post-branchial plexus fail to connect, the neighboring vasculature such as the ventral bud of the right 6th aortic arch may provide the needed blood supply to the developing lung and result in an anomalous LPA originating from the RPA [27]. If such a connection occurs dorsal or ventral to the trachea, the anomalous LPA develops anteriorly or posteriorly to the tracheobronchial tree, respectively [18]. Embryologically, the pulmonary artery sling develops when the left lung bud fails to connect with the left sixth arch and instead forms a connection with the right sixth arch dorsal to the developing lung bud, which results in LPA arising from the RPA and coursing posterior to the trachea. In the case of a partial LPA sling (PALPA “sling”), the anomalous LPA courses behind the trachea because of a dorsal connection to the lung bud and can result in symptoms such as stridor or shortness of breath. PALPA on the other hand occurs if such left lung bud forms a connection with the right sixth arch ventral to the developing lung bud with resultant LPA passing anterior to the bronchi [6]. The exact embryologic differences between the origins of a partial versus complete anomalous LPA and the interplay of these pulmonary artery anomalies with the other well documented cardiopulmonary and non-cardiopulmonary defects common in this patient population remains to be elucidated. 

In the present study, respiratory symptoms were reported in over half (60%) of patients with a posterior PALPA course which was a statistically significant difference when compared to the 20% of anterior PALPA patients reporting such symptoms, although no significant difference was found between presence of tracheobronchial anomalies. These findings suggest that symptoms may potentially be related to a dynamic narrowing of the tracheobronchial tree, which may not be seen without imaging. Dynamic evaluation of PALPA anomalies using ultrasound or CT with careful attention to the relationship between the anomaly and tracheobronchial tree during the respiratory cycle may be a worthwhile area of further research. To our knowledge, this is the first reported instance of a significant clinical difference between PALPA subtypes. Awareness of PALPA course may provide the treating team with an ability to anticipate and triage those patients at higher risk of developing respiratory symptoms prior to presentation or even antenatally.

In an autopsy series of 68 LPAS patients, 40% were found to have tracheobronchial abnormalities including hypoplasia of the distal trachea (38%) with complete cartilaginous rings, stenosis of the left main bronchus (5%) and direct origin of the right upper lobe bronchus from the trachea (bronchus suis, 12%) [29]. Major congenital cardiovascular anomalies were reported in 30% of these patients [29]. Most reported cases of PALPA have been in the pediatric population, although cases in the adult population have been diagnosed [1,11]. Excluding the 28-year-old patient with a history of TOF repair reported by Mathias et al. [11], the other two adult patients in our series were diagnosed incidentally with no respiratory symptoms or association with other cardiovascular, tracheobronchial or non-cardiopulmonary defects.

Bridging bronchus represents a rare congenital anomaly in which the right lower lobe and occasionally the right middle lobe ventilation is supplied by an aberrant bronchus arising from the left main bronchus crossing the mediastinum while the right upper lobe is supplied by the right main bronchus. It is commonly accompanied by a long or short segment of tracheal and/or bronchial stenosis [30]. Affected patients typically present with signs of severe airway obstruction. This is commonly associated with cardiovascular anomalies including LPAS, PALPA, tetralogy of Fallot, aortic coarctation and more [29]. Interestingly enough are the similarities of anomalies between LPAS and PALPA. Wells et al., in a systematic review of LPAS patients, identified two different tracheobronchial patterns [31]: Type 1, with a normal tracheobronchial tree with airway compression from the aberrant left pulmonary artery and Type 2, the most common pattern, which can be divided into two sub-patterns, 2A with a bridging bronchus, and 2B, with absence of the right bronchial tree and a commonly hypoplastic right lung, supplied by a bronchial branch from the left bronchus [31].

In this series, there was a high prevalence of congenital heart disease with a broad spectrum of conditions ranging from no additional cardiovascular anomalies to complex multiple additional cardiovascular anomalies. Similarly, in a series of patients with LPAS, Xie et al. reported 85% of patients were associated with congenital heart disease, with ventricular septal defects (47%), atrial septal defects (43%), patent ductus arteriosus (34%) and persistent left superior vena cava (30%) being the most common [4].

Patients with a diagnosis of Kabuki syndrome have been reported to have some type of congenital heart disease in 60 to 90% of cases, in particular coarctation of the aorta (23%-46%), atrial septal defect (20%) and ventricular septal defect (17%) [5,32]. This strong association was also found in 17% of patients in this series. Overall, patients with diagnosed or suspected Kabuki syndrome in this study were associated with relatively simple additional cardiovascular anomalies besides PALPA; this suggests the possibility of a potentially different embryologic origin when compared with the non-syndromic cases which tended to have multiple additional cardiovascular anomalies other than PALPA. It is worth mentioning that due to its extreme rarity, it is possible that the overall prevalence of Kabuki syndrome in this patient population may be underestimated.

This study is limited by its retrospective nature and the limited number of published literature on the topic related to the exceedingly rare nature of the condition and relatively new technology of cross sectional imaging. It is important to highlight that this work is limited by the exclusive evaluation of symptoms related to the respiratory system and did not assess for non-respiratory symptomatology. Additionally, not all published cases had all the information that was being investigated as some cases were published singularly as rare, interesting cases, while others were presented as single-center series. Lastly, the grouping together of anterior and anterior–inferior coursing PALPA did not allow for the statistical discrimination between these two PALPA subtypes, although their close morphology makes for any clinical differences to be probably unlikely. Notwithstanding, the results presently discussed are derived from what is to our knowledge the largest collection of patients with PALPA to date and helps gain new insights into this rare but clinically relevant entity. 

## 5. Conclusions

PALPA is a complex condition characterized by its anterior or posterior course about the tracheobronchial tree. This anomaly is most often diagnosed in childhood, is commonly associated with congenital heart disease, tracheobronchial anomalies and extra-thoracic abnormalities, most often feeds the left lower lung and may be associated with complex genetic syndromes. Of the analyzed variables, the only statistically significant finding was that respiratory symptoms were more common in posterior PALPA, possibly due to a dynamic narrowing of the tracheobronchial tree by a sling morphology. Additionally, our study demonstrates that posterior PALPA is more common than previously thought.

## Figures and Tables

**Table 1:** Comprehensive table of all cases obtained from the literature (*n* = 33) and the teaching institution (*n* = 2).

Case		Reference	Age	PALPA Relation to TB Tree	Feeding Pulmonary Segment	TB Tree Anomalies	Respiratory Issues (YN)	CVD OT PALPA M/S/N	Other Anomaly
1	M	New case	10 d	Anterior	?	N	N	HLHS, DORV, CoA	
2	M	New case	51 y	Anterior	?	N	N	N	
3	M	Erickson, 1996 [6]	30 m	Anterior	LLL	N	N	VSD	Hypospadias
4	M	Koch, 2000 [7]	5 m	Anterior	LLL	N	N	CoA, MS	Imperforate anus
5	N/A	Fountain-Dommer, 2001 [8]	Newborn	Anterior	LLL	N	N	HLHS, DORV, VSD, CoA, L sided A arch, R subclavian from descending aorta	
6	N/A	Fountain-Dommer, 2001 [8]	19 d	Anterior	LLL	N	N	CoA	
7	N/A	Divekar, 2004 [9]	Newborn	Anterior	LLL	N	N	TOF, TAPVR	
8	F	Collins, 2009 [10]	48 m **	Anterior	LLL	LMB stenosis	N	ASD	Skeletal malformations
9	M	Mathias, 2012 [11]	28 y	Anterior	LLL	RTB, R main bronchial diverticulum	N	TOF	
10	M	Bhat, 2012 [12]	Newborn **	Anterior	LLL	N	Y (“issues with airway”)	CoA	Dysmorphic features, maxillary teeth, undescended testes
11	N/A	Giudici, 2016 [13]	84 m **	Anterior	LLL	N	N	MS	SLE, small left kidney, hypothyroidism, GERD
12	F	Wang, 2017 [3]	9 m	Anterior	LUL	RTB	N	PDA	
13	M	Wang, 2017 [3]	4 m **	Anterior	LLL	LMB stenosis	Y (cough)	CoA, MS, MR	
14	M	Wang, 2017 [3]	38 m	Anterior	LLL	N	N	DORV, VSD, PS, SA, TAPVC	
15	F	Wang, 2017 [3]	2 m	Anterior	LLL	N	N	VSD, ASD	
16	F	Wang, 2017 [3]	40 m	Anterior	LUL	Mild trachea stenosis	Y (cough)	PA, VSD, RAoA	
17	M	Wang, 2017 [3]	18 m	Anterior	LLL	N	N	CoA, PDA, VSD	
18	F	Wang, 2017 [3]	27 m	Anterior	LLL	RTB, mild LMB stenosis	Y (SOB)	TOF, LSVC, PDA	
19	M	Duong, 2018 [14]	11 m	Anterior	LLL	N	N	VSD	
20	F	Chen, 2020 [15]	8 m **	Anterior	LUL	LMB stenosis	N	N	Facial anomaly, low set ears, funnel chest
21	N/A	Ge, 2001 [16]	2 m	Posterior	LLL	N	N	DORV, VSD, PS, MS, ASD, Hypoplastic LV	
22	N/A	Sadagopan, 2008 [17]	36 w	Posterior	LLL	N	N	VSD, CoA	Pelvicalyceal dilatation of kidneys, dysmorphic features (micrognathia, clinodactyly)
23	M	Tissot, 2008 [18]	Newborn	Posterior	LLL	N	Y (inc. work of breathing)	Swiss cheese VSD, PDA	Imperforate anus, cleft palate, two neonatal teeth, dysmorphic facies, small right pelvic kidney, left hydronephrosis, hypothyroidism
24	N/A	Tateishi, 2009 [19]	4 m	Posterior	LLL	TS	Y (dyspnea)	“AV septal defect with regurg”	
25	F	Collell, 2010 [20]	5 y	Posterior	LLL	Yes; unspecified	Y (stridor)	N	
26	F	Abelardo, 2013 [21]	3 m	Posterior	LUL	BB, LSTBS (Christmas tree)	Y (stridor)	N	Imperforate anus, rectovaginal fistula
27	M	Sen, 2015 [2]	“Baby”	Posterior	LLL	N	N	ASD, MA, VSD, BAV, CoA	Anal atresia, T3 butterfly vertebra, left T4 hemivertebra
28	M	Sen, 2015 [2]	1 d	Posterior	LLL	N	N	TOF	CHARGE association
29	N/A	Giudici, 2016 [13]	36 m	Posterior	“Left lung”	RTB, BB (Christmas tree)	Y (pulmonary infections, cough)	ASD	
30	N/A	Giudici, 2016 [13]	48 m **	Posterior	“Left lung”	LMB stenosis	Y (pulmonary infections, pulmonary hypertension)	VSD	Microcephaly, cleft palate, developmental delay
31	F	Wang, 2017 [3]	5 m	Posterior	LLL	BB, LSTS	Y (cough, SOB)	N	
32	M	Wang, 2017 [3]	5 m	Posterior	LLL	RTB, LSTS	N (not reported)	N	
33	M	Nagatomo, 2017 [22]	16 y	Posterior	LLL	Dynamic TS, LTB	Y (dyspnea on exertion)	N	
34	F	Chao, 2018 [23]	1 d	Posterior	LLL and part of LUL	LMB stenosis, hypoplastic R lung	Y (severe respirator distress)	Scimitar, dextrocardia	T12 hemivertebra
35	M	Maldjian, 2020 [1]	72 y	Posterior	LUL	N	N	N	

Note: ** = Kabuki syndrome (suspected or diagnosed); N/A = Not Available; M = Male; F = Female; w = Weeks; d = Days; m = Months; y = Years; N = No; Y = Yes; MS = Mitral Stenosis; VSD = Ventricular Septal Defect; ASD = Atrial Septal Defect; PDA = Patent Ductus Arteriosus; CoA = Coarctation of Aorta; TOF = Tetralogy of Fallot; DORV = Double Outlet Right Ventricle; PS = Pulmonary Stenosis; PA = Pulmonary Atresia; TAPVC = Totally Anomalous Pulmonary Venous Connection; RAoA = Right Aortic Arch; LSVC = Left Superior Vena Cava; MA = Mitral Atresia; BAV = Bicuspid Aortic Valve; HLHS = Hypoplastic Left Heart Syndrome; TB = Tracheobronchial; CVD OT PALPA = Cardiovascular defect other than PALPA; Ca = Carina; LLLB = Left lower lobe bronchus; LMB = Left main bronchus; RMB = Right main bronchus; Rt = Right; Lt = Left; RTB = Right tracheal bronchus; BB = Bridging bronchus; LSTS = Long segment tracheal stenosis; LSTBS = Long segment Tracheobronchial stenosis; SLE = Systemic Lupus Erythematosus; CHARGE assoc. = Coloboma, heart defects, choanal atresia, growth retardation, genital abnormalities, ear abnormalities association.

## Data Availability

The data presented in this study are available on request from the corresponding author.
